# Morphometric changes after endoscopic third ventriculostomy: a pediatric—adult comparison

**DOI:** 10.1007/s00381-026-07166-3

**Published:** 2026-02-17

**Authors:** Dimitrios Emmanouilidis, Pawel Krukowski, Witold Polanski, Thomas Pinzer, Claudia Zinke, Sebastian Brenner, Stephan B. Sobottka, Ilker Y. Eyüpoglu

**Affiliations:** 1 Department of Neurosurgery, Division of Pediatric Neurosurgery, Faculty of Medicineand , University Hospital Carl Gustav Carus, Technische Universität Dresden, Dresden, Germany; 2 Department of Neuroradiology, Faculty of Medicine and, University Hospital Carl Gustav Carus, Technische Universität Dresden, Dresden, Germany; 3 Department Pediatrics, Pediatric Oncology, Faculty of Medicine and, University Hospital Carl Gustav Carus, Technische Universität Dresden, Dresden, Germany; 4 Department Pediatrics, Pediatric Intensive Care Medicine, Faculty of Medicineand , University Hospital Carl Gustav Carus, Technische Universität Dresden, Dresden, Germany

**Keywords:** Triventricular hydrocephalus, Mamillopontine distance, Evans’ index, Diameter of third ventricle

## Abstract

**Purpose:**

Endoscopic third ventriculostomy (ETV) is an established treatment for triventricular hydrocephalus (TVH) caused by aqueductal stenosis (AqS) or mass lesions and is also applied in longstanding overt ventriculomegaly of adults (LOVA). This study aimed to compare morphometric remodeling on magnetic resonance imaging (MRI) after ETV between pediatric and adult patients.

**Methods:**

This retrospective single-center study included 67 patients with TVH or LOVA (patent aqueduct), stratified into children with AqS (*n* = 21), adults with AqS (*n* = 24), adults with lesions (*n* = 12), and adults with LOVA (*n* = 10). Mamillopontine distance (MPD), Evans’ index (EI), and third ventricle diameter (D3V) were measured preoperatively, postoperatively, and at follow-up. Longitudinal changes were compared within and between groups.

**Results:**

Follow-up MRI was available in 88.1% (*n* = 59/67). No child required shunt placement, compared with 12.5% of adults with AqS, 8.3% with lesions, and 30% with LOVA. Morphometric analysis revealed significant improvement (*p* < 0.05) in MPD, EI, and D3V from baseline to follow-up in children and adults with AqS. Adults with lesions showed significant changes only in MPD and D3V, whereas adults with LOVA exhibited no significant differences. Between-group analyses confirmed the most distinct and consistent ventricular remodeling in pediatric patients.

**Conclusion:**

Children demonstrated the most pronounced morphometric response after ETV, reflecting more effective ventricular remodeling than in adults. These findings suggest a stronger cerebrospinal fluid dynamic adaptation in pediatric patients, which may contribute to more favorable long-term outcomes.

## Introduction

Endoscopic third ventriculostomy (ETV) is an established neurosurgical procedure for the treatment of triventricular hydrocephalus (TVH), a common form of obstructive hydrocephalus (ObH) in both children and adults [1–5]. A frequent cause of TVH is aqueductal stenosis (AqS), which may be congenital or secondary to mass lesions such as tectal glioma or pineal tumors [4, 6–8]. In addition, patients with longstanding overt ventriculomegaly in adults (LOVA)—a condition with debated pathogenesis and variable classification depending on aqueductal patency—are also commonly treated with ETV as a first-line therapy [9–14].

Despite the established role of ETV, the extent of postoperative ventricular remodeling, including long-term changes, remains insufficiently characterized. Furthermore, potential differences in radiological response patterns between pediatric and adult patients have not been systematically assessed.

In the present study, we retrospectively analyzed routine magnetic resonance imaging (MRI) data to evaluate three morphometric parameters: mamillopontine distance (MPD), Evans’ index (EI), and the diameter of the third ventricle (D3V). These parameters were chosen because they can be readily assessed in clinical practice and provide an immediate impression of hydrocephalus dynamics. This study aimed primarily to determine whether morphometric changes after ETV differ significantly between pediatric and adult patients. We hypothesized that pediatric patients would demonstrate more pronounced ventricular remodeling following ETV, reflecting a greater structural responsiveness compared with adults.

## Methods and materials

### Study design

This retrospective, single-center study included pediatric and adult patients treated for TVH or LOVA with a patent aqueduct. Patients with other indications for ETV were excluded. All patients underwent ETV at the Department of Neurosurgery, University Hospital Dresden. The surgical procedures were performed with a rigid endoscope, using either the LOTTA® ventriculoscope (Karl Storz) or the MINOP® system (B. Braun).

**Fig. 1:**
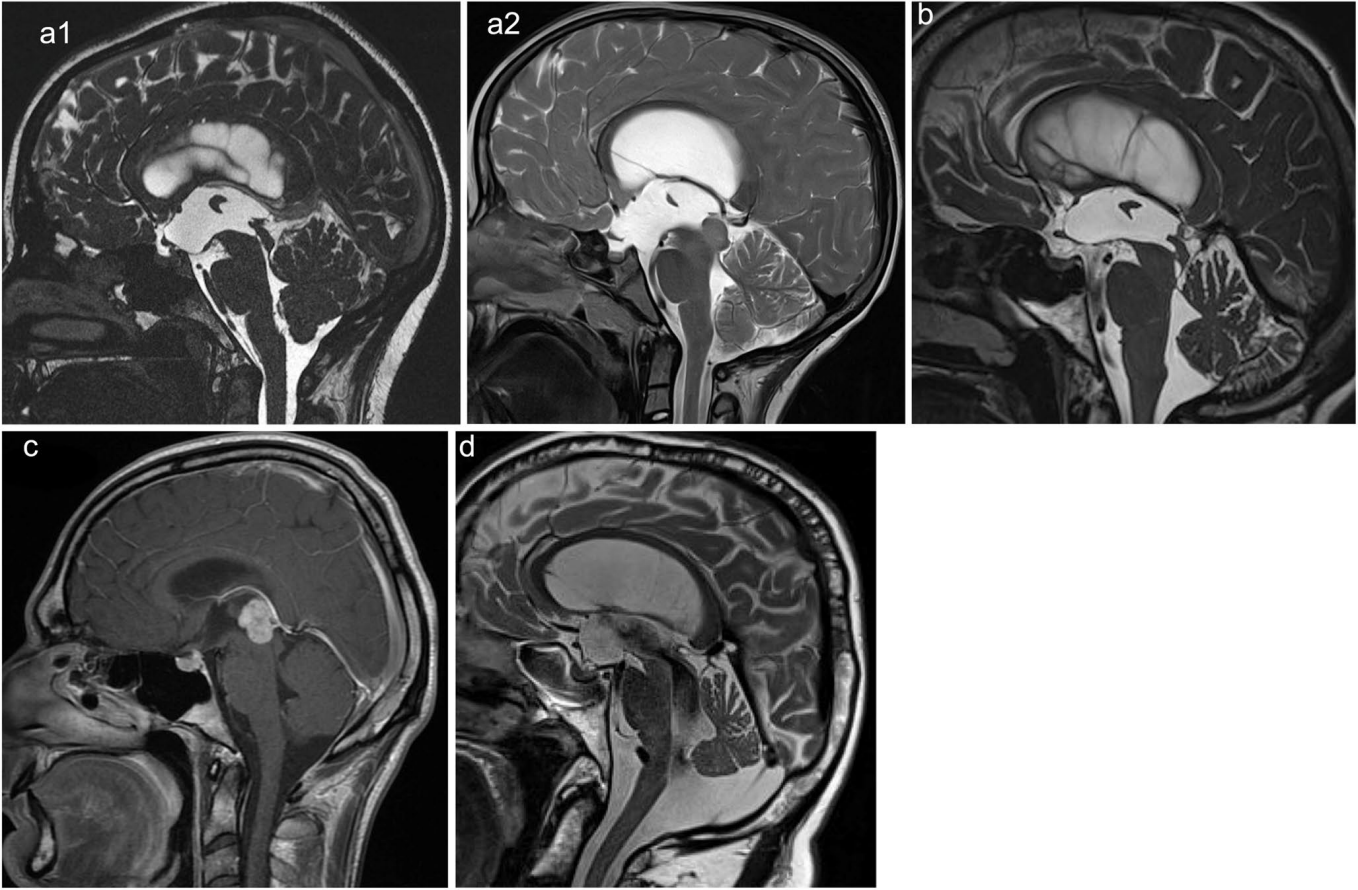
Representative preoperative sagittal MRI of each patient group. *AqS* aqueductal stenosis, *LOVA* longstanding overt ventriculomegaly in adults, *CISS* Constructive interference in steady state. **a1** Child with AqS (CISS sequence). **a2** Child with tectal tumor (T2-weighted). **b** Adult with AqS (T2-weighted). **c** Adult with lesion (T1-weighted with gadolinium). **d** Adult with LOVA (T2-weighted)

Patients were stratified into four groups based on age and underlying pathology (Fig. 1):

1. Children with AqS: pediatric patients with AqS, either congenital or secondary to tectal/mesencephalic tumors or pineal cysts.
2. Adults with AqS: adult patients with membranous AqS.
3. Adults with lesions: adult patients with TVH due to tectal/mesencephalic or pineal mass lesions.
4. Adults with LOVA: adult patients with LOVA and a patent aqueduct [13, 14].

All patients underwent at least one preoperative and one postoperative MRI, with the majority also receiving long-term follow-up imaging. Morphometric measurements were obtained preoperatively, immediately postoperatively, and at long-term follow-up. In this study, follow-up refers to the last available MRI examination.

### Morphometric analysis

All MRI measurements were performed by the same consultant neuroradiologist (P. K.).

The following parameters were evaluated:

- Mamillopontine distance: distance between the inferior margin of the mamillary bodies and the superior surface of the pons on sagittal MRI [15, 16].
- Evans’ index: ratio of the maximum width of the frontal horns of the lateral ventricles to the maximum internal skull diameter at the same axial level [17–19].
- Diameter of the third ventricle: maximum transverse diameter of the third ventricle on axial MRI [20, 21].

MPD, EI, and D3V were measured for each group at three time points: preoperatively, postoperatively (on the first or second postoperative day), and at follow-up. T2-weighted, fluid-attenuated inversion recovery (FLAIR), and constructive interference in steady state (CISS) sequences were used, with sagittal images applied for the measurement of MPD and axial images for the measurement of EI and D3V. To assess the efficacy of ETV, changes in these morphometric parameters were analyzed within each patient group across the defined time intervals, followed by intergroup comparisons. In addition, the patency of the stoma in the floor of the third ventricle was assessed on sagittal T2-weighted and CISS sequences as a supplementary indicator of ETV efficacy.

All measurements of MPD, EI, and D3V were obtained exclusively from MRI examinations performed before and after ETV. No imaging acquired after ventriculoperitoneal shunt (VP shunt) implantation was included in the analysis.

### Clinical data

Demographic data, diagnoses, and the need for subsequent VP shunt implantation after ETV were extracted from electronic hospital records during inpatient treatment and outpatient follow-up visits.

### Ethics

The study was approved by the local Ethics Committee of the Technische Universität Dresden (approval number: BO ff (Mono)-EK-211052025).

### Statistical analysis

Quantitative variables were expressed as mean ± standard deviation (SD) and as median with interquartile range (IQR), while categorical variables were expressed as absolute and relative frequencies. Normality of quantitative variables was tested using the Kolmogorov–Smirnov test. Follow-up time was compared among the four groups using the Kruskal–Wallis test. The proportions of patients requiring VP shunt implantation were compared using Fisher’s exact test. Repeated measures analysis of variance (ANOVA) was performed to evaluate changes in MPD, EI, and D3V within each group over the follow-up period, as well as to compare the degree of change between groups. Bonferroni correction was used in cases of multiple testing to control for type I error. All reported p values are two-tailed, and statistical significance was set at *p* < 0.05. Analyses were conducted using SPSS statistical software (version 27.0).

## Results

### Study population

The study population comprised 67 patients. Of these, 21 were children (< 18 years), all classified as children with AqS. A total of 46 adults were included and divided into three groups: 24 adults with AqS, 12 adults with lesions, and 10 adults with LOVA (Table 1).

**Table 1:** Age at surgery, time of follow-up and VP shunt

Group	Age at first ETV (years)			Follow-up period (months)			VP shunt
	Range	Mean (SD)	Median (IQR)	Range	Mean (SD)	Median (IQR)	*n* (%)
Children with AqS (*n* = 21)	0.2–17.3	8.7 (6.1)	10.0 (1.8–13.2)	2.8–96.9	31.5 (27.9)	21.9 (9.6–52)	0.0 (0.0)
Adults (*n* = 46)							
-with AqS (*n* = 24)	22.2–76.6	55.6 (16.3)	59.5 (45.8–67.9)	1.4–101.7	12.1 (21.2)	6.8 (3.1–9.5)	3.0 (12.5)
-with lesions (*n* = 12)	22.8–80.7	53.0 (19.4)	55.8 (38.8–67.8)	1.5–48.1	14.7 (16.7)	7.4 (4.2–20.0)	1.0 (8.3)
-with LOVA (*n* = 10)	54.2–75.1	64.7 (6.9)	63.7 (60.6–69.1)	0.6–39.7	9.3 (12.3)	3.3 (2.6–9.5)	3.0 (30.0)
*P* value					**0.005**^a, b^		0.068^c^

*SD* standard deviation, *IQR* interquartile range, *AqS* aqueductal stenosis, *LOVA* longstanding overt ventriculomegaly in adults, *ETV* endoscopic third ventriculostomy, *VP* shunt ventriculoperitoneal shunt

^a^Kruskal-Wallis test

^b^After Bonferroni correction: follow-up duration in “children with AqS” was significantly longer compared with “adults with AqS” (*p* = 0.010) and “adults with LOVA” (*p *= 0.025)

^c^Fisher’s exact test

The proportion of male patients was 52.4% (*n* = 11/21) in children with AqS, 37.5% (*n* = 9/24) in adults with AqS, 25.0% (*n* = 3/12) in adults with lesions, and 30.0% (*n* = 3/10) in adults with LOVA.

The time frames of the ETV procedures were July 2013 to January 2025 in children, September 2012 to November 2024 in adults with AqS, July 2013 to May 2024 in adults with lesions, and July 2013 to March 2025 in adults with LOVA.

In children, the cause of AqS was congenital in 57.1% (*n* = 12/21) and acquired in 42.9% (*n* = 9/21). Among acquired cases, 88.9% (*n* = 8/9) were due to tectal/mesencephalic tumors and 11.1% (*n* = 1/9) to a pineal cyst.

In adults with lesions, the underlying pathology was a tectal/mesencephalic tumor in 50.0% (*n* = 6/12), pineal metastasis in 33.3% (*n* = 4/12), and pineal germ cell tumor and pineal cyst in 8.3% (*n* = 1/12) of cases, respectively.

### Age at first ETV

In children, the mean age was 8.7 years (SD 6.1; range 0.2–17.3). Among adults, it was 55.6 years (SD 16.3; range 22.2–76.6) in the group with AqS, 53.0 years (SD 19.4; range 22.8–80.7) in adults with lesions, and 64.7 years (SD 6.9; range 54.2–75.1) in adults with LOVA (Table 1).

### Follow-up period

Follow-up MRI was available in 90.5% (*n* = 19/21) of children, 91.6% (*n* = 22/24) of adults with AqS, 75.0% (*n* = 9/12) of adults with lesions, and 90.0% (*n* = 9/10) of adults with LOVA, corresponding to an overall follow-up rate of 88.1% (*n* = 59/67).

The mean MRI follow-up duration was 31.5 months (SD 27.9; range 2.8–96.9) in children with AqS, 12.1 months (SD 21.2; range 1.4–101.7) in adults with AqS, 14.7 months (SD 16.7; range 1.5–48.1) in adults with lesions, and 9.3 months (SD 12.3; range 0.6–39.7) in adults with LOVA. The follow-up period differed significantly among the four groups (*p* = 0.005). After Bonferroni correction, it was significantly longer in children with AqS compared with adults with AqS (*p* = 0.010) and adults with LOVA (*p* = 0.025) (Table 1).

### Implantation of VP shunt

None of the pediatric patients required VP shunt implantation after ETV. In contrast, shunt implantation was necessary in 12.5% (*n* = 3/24) of adults with AqS, 8.3% (*n* = 1/12) of adults with lesions, and 30% (*n* = 3/10) of adults with LOVA due to persistent symptoms. The proportion of VP shunt implantation showed a trend toward differences among the groups (*p* = 0.068) (Table 1).

A second ETV was required in one pediatric patient and was not performed in adults.

### Morphometric parameters

#### Mamillopontine distance

MPD values preoperatively, postoperatively, and at follow-up, as well as their within-group changes, are summarized in Table 2.

Preoperatively, groups differed significantly in baseline MPD. Children with AqS had smaller mean MPD compared with adults with AqS (*p* = 0.039) and adults with LOVA (*p* = 0.023). No other significant differences were observed between groups at baseline. Postoperatively and at follow-up, absolute MPD values did not differ significantly among the four groups (Table 2).

Within-group analyses revealed marked time-dependent changes. In children with AqS mean MPD increased continuously from 4.09 mm preoperatively to 6.91 mm at follow-up, with all pairwise comparisons (preoperatively-postoperatively, postoperatively-follow-up, preoperatively-follow-up) remaining significant (*p* < 0.001). Significant increases across time points were also observed in adults with AqS and in adults with lesions (*p* < 0.05). In contrast, MPD in adults with LOVA remained largely stable, without significant changes (Table 2).

**Fig. 2:**
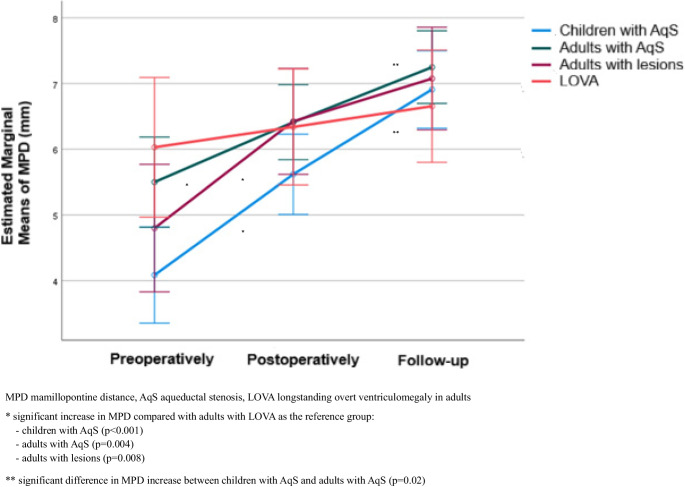
Longitudinal changes in MPD, stratified by group

A significant difference in MPD time courses between groups was confirmed (*p* < 0.001), indicating that MPD increased over time overall but with distinct longitudinal patterns (Table 2, Fig. 2). More specifically, the degree of MPD increase was greater in children with AqS (*p* < 0.001), adults with AqS (*p* = 0.004), and adults with lesions (*p* = 0.008) when compared with adults with LOVA. In addition, the increase was significant greater in children with AqS than in adults with AqS (*p* = 0.02) (Fig. 2).

**Table 2:** MPD values and changes over time

MPD (mm)	Group							
	Children with AqS (a)		Adults with AqS (b)		Adults with lesions (c)		Adults with LOVA (d)	
	Mean (SD)	Median (IQR)	Mean (SD)	Median (IQR)	Mean (SD)	Median (IQR)	Mean (SD)	Median (IQR)
Preoperatively	4.09 (1.61)^b,d^	4.0 (3.0–4.5)	5.50 (1.70)^a^	5.3 (4–7.15)	4.80 (1.66)	5.4 (3.75–6.1)	6.03 (1.80)^a^	5.75 (5.3–6.6)
Postoperatively	5.62 (1.03)	5.8 (5.0–6.2)	6.41 (1.58)	6.35 (5.2–7.4)	6.43 (1.17)	6.35 (5.85–7.15)	6.34 (1.82)	5.95 (5.3–6.6)
Follow-up	6.91 (1.27)	6.91 (6.2–7.5)	7.25 (1.42)	7.33 (6.2–8.15)	7.08 (1.02)	7.08 (7–7.25)	6.66 (1.68)	6.35 (5.4–7.3)
Changes:								
Postoperatively – Preoperatively	1.53 (1.12)	1.8 (1–2)	0.91 (0.89)	0.8 (0.2–1.3)	1.63 (1.46)	1.35 (0.25–2.9)	0.31 (0.52)	0.1 (0.0–0.2)
P^1^	**<0.001**		**<0.001**		**<0.001**		>0.999	
Follow-up – Postoperatively	1.29 (1.12)	1.1 (0.4–1.9)	0.84 (0.82)	0.65 (0.15–1.3)	0.65 (0.82)	0.89 (0.1–1.3)	0.32 (0.37)	0.3 (0.06–0.5)
P^1^	**<0.001**		**<0.001**		**0.039**		0.786	
Follow-up – Preoperatively	2.83 (1.79)	3.4 (2–3.91)	1.75 (1.12)	1.7 (0.8–2.35)	2.28 (1.7)	1.53 (0.99–3.45)	0.63 (0.66)	0.4 (0.16–0.8)
P^1^	**<0.001**		**<0.001**		**<0.001**		0.514	
P^2^	**<0.001**							

*MPD* mamillopontine distance, *AqS* aqueductal stenosis, *LOVA* longstanding overt ventriculomegaly in adults, *SD* standard deviation, *IQR* interquartile range

a, b, c, d they indicate significant between-group differences (after Bonferroni correction)

P^1^ significance of within-group changes across time (after Bonferroni correction)

P^2^ significance of group differences in time-course (repeated measures ANOVA)

#### Evans’ index

EI values and their evolution over time are summarized in Table 3.

Across all patient groups, mean EI at each time point exceeded 0.3, consistent with ventriculomegaly. After Bonferroni correction, adults with LOVA had consistently higher EI compared with all other groups (*p* < 0.05). In addition, preoperatively, children with AqS exhibited greater EI than adults with lesions (*p* = 0.019) (Table 3).

Distinct temporal trends emerged within patient´s groups. In children with AqS, EI decreased significantly between all time points (preoperatively-postoperatively: *p* = 0.006, postoperatively-follow-up: *p* < 0.001, preoperatively-follow-up: *p* < 0.001). In adults with AqS, a mild but significant reduction was noted only between baseline and follow-up (*p* = 0.004). EI in adults with lesions and adults with LOVA remained stable without significant change (Table 3).

**Fig. 3:**
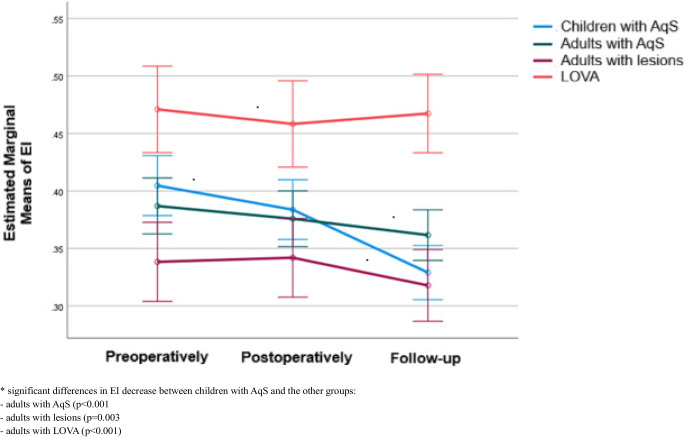
Longitudinal changes in EI, stratified by group

Group comparison (repeated measures ANOVA) confirmed significant heterogeneity (*p* < 0.001), with the most pronounced reduction in children with AqS (Table 3, Fig. 3). Pairwise analyses demonstrated a greater EI decrease in children with AqS compared with adults with AqS (*p* < 0.001), adults with lesions (*p* = 0.003), and adults with LOVA (*p* < 0.001). By contrast, the degree of reduction did not differ significantly among the adult groups (Fig. 3).

**Table 3:** EI values and changes over time

EI	Group							
	Children with AqS (a)		Adults with AqS (b)		Adults with lesions (c)		Adults with LOVA (d)	
	Mean (SD)	Median (IQR)	Mean (SD)	Median (IQR)	Mean (SD)	Median (IQR)	Mean (SD)	Median (IQR)
Preoperatively	0.40 (0.07)^c,d^	0.39 (0.37–0.45)	0.39 (0.05)^d^	0.38 (0.36–0.42)	0.34 (0.03)^a,d^	0.33 (0.32–0.36)	0.47 (0.09)^a,b,c^	0.45 (0.4–0.53)
Postoperatively	0.38 (0.07)^d^	0.38 (0.34–0.42)	0.38 (0.05)^d^	0.37 (0.33–0.42)	0.34 (0.05)^d^	0.35 (0.31─0.36)	0.46 (0.07)^a,b,c^	0.44 (0.4–0.51)
Follow-up	0.33 (0.06)^d^	0.33 (0.29–0.37)	0.36 (0.04)^d^	0.36 (0.33─0.39)	0.32 (0.01)^d^	0.32 (0.31─0.33)	0.47 (0.08)^a,b,c^	0.46 (0.41–0.53)
Changes:								
Postoperatively – Preoperatively	−0.02 (0.03)	−0.01 (−0.04 – −0.01)	−0.01 (0.02)	−0.02 (−0.03 – 0.00)	0.00 (0.05)	0.00 (−0.02 – 0.02)	−0.01 (0.02)	−0.02 (−0.02– 0.00)
P^1^	**0.006**		0.223		>0.999		0.559	
Follow-up – Postoperatively	−0.05 (0.05)	−0.05 (−0.07 – −0.03)	−0.01 (0.03)	−0.02 (−0.03 – 0.00)	−0.02 (0.05)	−0.02 (−0.05 – 0.02)	0.01 (0.04)	0.01 (−0.01 – 0.03)
P^1^	**<0.001**		0.278		0.134		>0.999	
Follow-up – Preoperatively	−0.08 (0.05)	−0.07 (−0.11 – −0.04)	−0.03 (0.03)	−0.03 (−0.05 – −0.01)	−0.02 (0.03)	−0.03 (−0.04 – 0.00)	0.00 (0.03)	−0.01 (−0.02 – 0.00)
P^1^	**<0.001**		**0.004**		0.169		>0.999	
P^2^	**<0.001**							

*EI* Evan’s index, *AqS* aqueductal stenosis, *LOVA* longstanding overt ventriculomegaly in adults, *SD* standard deviation, *IQR* interquartile range

a, b, c, d they indicate significant between-group differences (after Bonferroni correction)

P^1^ significance of within-group changes across time (after Bonferroni correction)

P^2^ significance of group differences in time-course (repeated measures ANOVA)

#### Diameter of the third ventricle

The changes in D3V values throughout follow-up are presented in Table 4 for each group separately.

Significant intergroup differences were detected after Bonferroni correction. Adults with LOVA had consistently higher values compared with all other groups (*p* < 0.05). At follow-up, children with AqS had significantly lower D3V values than adults with AqS (*p* = 0.004). At all time points, children presented with the lowest mean D3V (Table 4).

Over time, divergent patterns emerged within the groups. In children with AqS, D3V decreased significantly from postoperative to follow-up (*p* < 0.001) and from preoperative to follow-up (*p* < 0.001), whereas no significant difference was seen between preoperative and postoperative measurements. Adults with lesions demonstrated a similar course. In adults with AqS, a significant reduction was already evident postoperatively compared with preoperative values (*p* < 0.001) and persisted at follow-up (*p* < 0.001). In contrast, D3V in adults with LOVA remained unchanged throughout the observation period (Table 4).

**Fig. 4:**
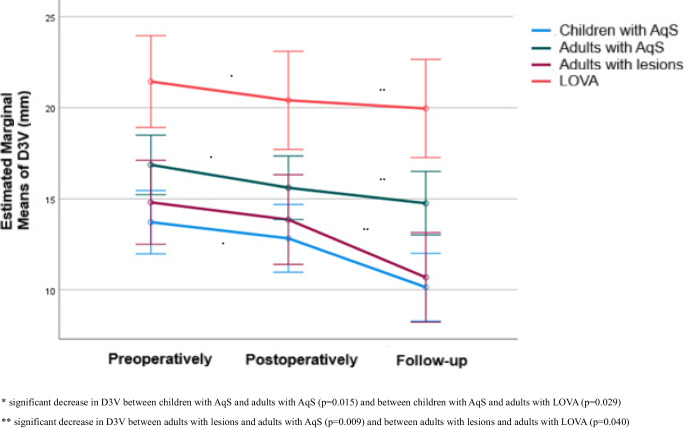
Longitudinal changes in D3V, stratified by group

When the extent of change was compared across groups (*p* = 0.014), the reduction was more pronounced in children with AqS than in adults with AqS (*p* = 0.015) and adults with LOVA (*p* = 0.029). Likewise, adults with lesions showed a greater decrease compared with both adults with AqS (*p* = 0.009) and adults with LOVA (*p* = 0.040) (Table 4, Fig. 4).

**Table 4:** D3V values and changes over time

D3V (mm)	Group							
	Children with AqS (a)		Adults with AqS (b)		Adults with lesions (c)		Adults with LOVA (d)	
	Mean (SD)	Median (IQR)	Mean (SD)	Median (IQR)	Mean (SD)	Median (IQR)	Mean (SD)	Median (IQR)
Preoperatively	13.72 (4.22)^d^	13.00(11.50–170)	16.86 (3.61)^d^	16.85(14–19)	14.81 (4.11)^d^	14.65(12.15–17.30)	21.44 (4.25)^a,b,c^	20.50(18.00–25.00)
Postoperatively	12.83 (4.89)^d^	12.00(9.50–14.80)	15.61 (3.60)^d^	15.4(12.85–17.15)	13.86 (4.01)^d^	13.85(10.50–15.40)	20.41 (4.64)^a,b,c^	20.25(17.00–24.20)
Follow-up	10.14 (5.35)^b,d^	10.00(7.00–14.10)	14.76 (3.91)^a,d^	14.65(12.15–17)	10.68 (1.83)^d^	10.70(9.30–11.95)	19.96 (4.56)^a,b,c^	19.15(16.00–24.20)
Changes:								
Postoperatively – Preoperatively	−0.89 (2.48)	−1.20(−2.00 – −0.40)	−1.25(1.07)	−1.3(−2.05 – −0.45)	−0.95(1.85)	−0.80(−1.95 – −0.40)	−1.03(0.90)	−1.05(−1.90 – −0.10)
P^1^	0.070		**0.003**		0.197		0.205	
Follow-up – Postoperatively	−2.69 (2.83)	−2.00(−4.80 – −1.40)	−0.85 (1.66)	−0.10(−1.60 – 0.00)	−3.18 (3.78)	−2.90(−4.80 – −0.55)	−0.45(1.71)	−0.40(−2.00 – 1.50)
P^1^	**<0.001**		0.318		**<0.001**		>0.999	
Follow-up – Preoperatively	−3.58 (2.81)	−3.90(−4.10 – −2.10)	−2.10 (1.98)	−1.20(−3.35 – −0.75)	−4.13 (3.57)	−4.20(−4.60 – −1.85)	−1.48(1.30)	−1.40(−2.50 – −0.50)
P^1^	**<0.001**		**<0.001**		**<0.001**		0.207	
P^2^	**0.014**							

*Significant decrease in D3V between children with AqS and adults with AqS (*p* = 0.015) and between children with AqS and adults with LOVA (*p* = 0.029)

**Significant decrease in D3V between adults with lesions and adults with AqS (*p* = 0.009) and between adults with lesions and adults with LOVA (*p* = 0.040)

## Discussion

In this study, the morphometric parameters MPD, EI, and D3V were assessed on MRI and compared between pediatric patients with TVH, adult patients with TVH, and patients with LOVA with a patent aqueduct. To minimize the confounding influence of age-dependent anatomical differences between children and adults, the analysis focused not only on absolute values but primarily on changes in MPD, EI, and D3V over time. These parameters were chosen because they represent simple, robust, and reproducible measurements that can be obtained reliably in routine clinical practice without the need for specialized software or advanced imaging tools.

The study hypothesis was that pediatric patients benefit the most from ETV with respect to changes in MPD, EI, and D3V.

Regarding MPD, previous studies have reported reduced values in hydrocephalus [22]. In ObH, MPD is even more reduced compared to other forms, demonstrating high specificity and sensitivity for its diagnosis [15, 23]. In normal adults, mean MPD has been measured at 7.0 mm (SD ± 1.3) [16]. In our cohort, mean MPD was below 7.0 mm preoperatively and immediately postoperatively across all groups. At follow-up, MPD exceeded 7.0 mm in adults with AqS and adults with lesions, reaching values consistent with reported normal ranges in adults. In children with AqS, a significant increase was observed at follow-up, reaching a mean of 6.91 mm, which is notable given the smaller anatomical dimensions in childhood (Table 2). Preoperatively, MPD in pediatric patients was significantly smaller than in adults with AqS and adults with LOVA, possibly suggesting a more aggressive course of ObH in children compared to adults, who may present with longer-standing, partially compensated hydrocephalus. A significant increase in MPD was observed postoperatively in all groups except in adults with LOVA. The steepest progression occurred in pediatric patients with AqS and was significantly greater compared to adults with AqS (Table 2, Fig. 2).

Concerning EI, values in all patient groups remained above 0.3 at all time points, indicating persistent ventriculomegaly [18, 19]. The highest EI values were consistently observed in patients with LOVA, reflecting the absence of significant changes in intracranial cerebrospinal fluid (CSF) spaces despite ETV (Table 3). In the literature, EI has been reported to decrease with age in normal children [24]. In our cohort, the group of children with AqS showed a significant decrease of EI throughout follow-up compared with all adult groups (Table 3, Fig. 3). This finding highlights the marked efficacy of ETV on CSF dynamics in children, which may secondarily provide improved conditions for neurodevelopment, encompassing cognitive, motor, and other functional domains [25–30].

According to previous studies, D3V increases with age [21, 31, 32]. One MRI morphometric study in healthy adults reported a mean third ventricle width of 3.37 mm [33]. In our cohort, all adult patients, regardless of group, exhibited markedly higher D3V values than this reference, reflecting hydrocephalus (Table 4). Overall, the lowest D3V values were observed in pediatric patients, whereas patients with LOVA showed the highest values. During follow-up after ETV, a significant decrease of D3V was observed in pediatric patients, adults with AqS, and adults with lesions, but not in adults with LOVA (Table 4). Pediatric patients and adults with lesions demonstrated the greatest reduction in D3V throughout follow-up compared with adults with AqS and adults with LOVA (Fig. 4). This similar response pattern may be explained by the more acute or short-standing nature of hydrocephalus in these groups, in contrast to the more chronic disease course in adults with AqS and particularly in adults with LOVA. This finding illustrates the capacity for morphological adaptability of the third ventricle after ETV, which may be of particular benefit in pediatric patients given the highly eloquent anatomical structures surrounding the third ventricle.

In patients with LOVA, no significant changes in the MPD, EI, or D3V were observed after ETV throughout follow-up (Tables 2, 3, and 4), indicating a stable ventricular configuration despite the procedure. Consequently, in LOVA, assessment of ETV efficacy should rely primarily on clinical status rather than on morphometric changes alone. In contrast, children with AqS showed significant changes in all three analyzed morphometric parameters from baseline to follow-up, reflecting a trend toward restoration of more physiological ventricular conditions after ETV (Tables 2, 3, and 4, Figs. 2, 3, and 4). The postoperative increase in MPD together with the reduction in EI and D3V corresponds to the physiological, age-related decrease in the ratio between ventricular volume and cerebral parenchyma observed in healthy children [34]. This highlights the high potential of pediatric patients to regain a normal developmental trajectory after ETV. Significant improvements in morphometric parameters from baseline to follow-up were also observed in adults with AqS and in adults with lesions (Tables 2, 3, and 4, Figs. 2, 3, and 4). Overall, a more dynamic change was evident in all groups with ObH due to congenital or acquired AqS compared with the patent aqueduct in patients with LOVA, characterized by long-standing ventricular dilation.

Ventricular dynamic adaptation after ETV can be understood as a gradual establishment of more physiological intracranial hydrodynamics and pressure–volume relationships, reflected by progressive morphometric changes over time. In pediatric patients, this adaptation appears to be facilitated by the high plasticity of the developing brain and ventricular system. Following ETV, normalization or reduction of chronically elevated intracranial pressure may allow ongoing brain growth to proceed under less adverse mechanical conditions, promoting gradual reshaping of supratentorial ventricular spaces. In addition, with closure of the fontanelles and cranial sutures and the transition of the skull from a compensatory to a rigid compartment, internal remodeling of the ventricular system may be further promoted once CSF pathways are restored, particularly in young children. Physiological shifts in ventricular-parenchymal proportions favoring cerebral parenchyma during normal maturation further support this concept [24, 34].

In adults, morphometric adaptation after ETV may be attenuated because brain and skull development are completed and tissue plasticity is reduced. Long-standing hydrocephalus states, including LOVA and chronic ObH, are frequently characterized by reduced cerebral compliance, limiting ventricular reversibility despite restoration of CSF pathways [35, 36]. Secondary pathologies prevalent in elderly patients, such as cerebral atrophy, microvascular disease, and neurodegenerative changes, may further reduce compliance and dampen morphometric responsiveness, contributing to a weaker association between radiological changes and clinical outcome in adult and long-standing hydrocephalus [13, 19, 37–41]. Despite these constraints, adults with AqS and adults with obstructive lesions still demonstrated significant morphometric improvement after ETV, albeit to a lesser extent than pediatric patients, indicating that meaningful ventricular remodeling remains possible in adult ObH.

Interpretation of these findings must consider methodological limitations. Imbalances in group sizes, particularly the relatively small LOVA subgroup, and differences in follow-up duration—most notably the shorter follow-up in LOVA—limit temporally equivalent assessment of long-term remodeling. In addition, rapid disease progression in some adults with mass lesions precluded long-term follow-up imaging. In our opinion, the extended observation period in pediatric patients, together with the consistent and progressive improvement in MPD, EI, and D3V, supports the observed age-related differences and suggests a more pronounced morphometric response to ETV in children.

The high efficacy of ETV in the pediatric cohort is further supported by the absence of secondary VP shunt implantation (Table 1). Age may have contributed to this favorable outcome, as only three children were younger than six months at surgery, an age group associated with higher ETV failure rates [3, 42–44]. In contrast, the highest rate of secondary VP shunt implantation was observed in patients with LOVA (30%), consistent with the literature recognizing both ETV and VP shunting as valid treatment strategies in this entity [10]. In our cohort, the decision for VP shunt implantation was driven primarily by clinical status rather than morphometric findings.

## Limitations

This study has several limitations.

Firstly, all morphometric measurements were performed by a single neuroradiologist, which may affect objectivity and reproducibility. As the measurements were carried out manually, accuracy could be compromised, particularly given that the parameters are defined in millimeters. In some cases, suboptimal MRI quality may have further influenced measurement precision. Independent assessment by an additional neuroradiologist could have improved reliability. Future implementation of AI-based systems may enable automated morphometric analysis with higher accuracy and reproducibility.

To minimize the impact of human measurement variability, we focused not on absolute values but on longitudinal changes in morphometric parameters. This approach was intended to yield the most representative and objective results possible.

Furthermore, an optimal approach would have included volumetric assessment of the ventricles. However, this is not always feasible, as it is time-consuming in routine clinical practice and often limited by financial constraints in hospitals worldwide. As an alternative, ventricular changes can be evaluated using linear measurements, as applied in our study with MPD, EI, and D3V [17, 45]. Although additional linear morphometric parameters have been described in the literature, these were not included in the current analysis.

The analysis was limited to morphometric changes and did not include a detailed evaluation of clinical status, which could have provided a more comprehensive assessment of ETV efficacy.

In addition, differences in follow-up duration between groups, as mentioned above, may have affected comparability. Although follow-up MRI was available in the majority of patients (88%, *n* = 59/67), not all patients underwent long-term imaging. In adults with mass lesions, the absence of follow-up MRI was primarily related to rapid disease progression and poor prognosis. In the remaining groups, missing follow-up imaging was mainly attributable to insufficient compliance by patients or caregivers.

Finally, the single-center design and relatively small sample size represent additional limitations, restricting the generalizability of the findings.

## Conclusion

ETV resulted in the greatest improvement in the morphometric parameters MPD, EI, and D3V from baseline to follow-up in pediatric patients with AqS compared with adult patients with TVH and LOVA, which may indicate a more effective restoration of CSF dynamics in children. The observed morphometric responsiveness in pediatric patients underscores the effectiveness of ETV in this age group and supports its use in achieving favorable outcomes.

## Data Availability

The data that support the findings of this study are not openly available due to reasons of sensitivity and are available from the corresponding author upon reasonable request.

## Abbreviations

*AqS*: Aqueductal Stenosis
*CISS*: Constructive Interference in Steady State
*CSF*: Cerebrospinal Fluid
*D3V*: Diameter of Third Ventricle
*EI*: Evans’ Index
*ETV*: Endoscopic Third Ventriculostomy
*FLAIR*: Fluid-attenuated inversion recovery
*IQR*: Interquartile Range
*LOVA*: Longstanding Overt Ventriculomegaly in Adults
*MPD*: Mamillopontine Distance
*MRI*: Magnetic Resonance Imaging
*ObH*: Obstructive Hydrocephalus
*SD*: Standard Deviation
*TVH*: Triventricular Hydrocephalus
*VP shunt*: Ventriculoperitoneal shunt
